# Lifestyles, genetics, and future perspectives on gastric cancer in east Asian populations

**DOI:** 10.1038/s10038-021-00960-8

**Published:** 2021-07-16

**Authors:** Hiroto Katoh, Shumpei Ishikawa

**Affiliations:** Department of Preventive Medicine, Graduate School of Medicine, The University of Tokyo, Tokyo, Japan

**Keywords:** Gastric cancer, Cancer genetics

## Abstract

The prevalence of gastric cancer (GC) differs among regions worldwide, with the highest occurrence in east Asia. Thus, its etiology, with respect to ethnic background, environmental factors, and lifestyles, is also thought to differ essentially. In addition, etiology of GC is speculated to be changing due to the recent decrease in the *Helicobacter pylori* (*H. pylori*) infection in Japan. State-of-the-art somatic/germline cancer genomics has clarified the etiologies of gastric carcinogenesis. In this review article, we summarize past and present milestones in our understanding of GC achieved through genomic approaches, including a recent report that revealed higher-than-expected frequencies of GCs attributed to east Asian-specific germline variants in *ALDH2* or *CDH1* in combination with lifestyles. Based on this updated knowledge, we also discuss the possible impact of and high-risk approaches for GCs in the upcoming “*H. pylori*-negative era.”

## Introduction

Gastric cancer (GC) is the fifth most frequently diagnosed malignancy and the third leading cause of cancer mortality worldwide [1]. However, the incidence of GC varies substantially around the globe and is the highest in east Asian countries including Japan, Korea, and China [1, 2]. In recent statistics in Japan, GC was reported to be the second and fourth most common cancer in men and women, respectively (https://ganjoho.jp/reg_stat/statistics/stat/summary.html). The most popular etiology of GC is *Helicobacter pylori* (*H. pylori*) infection. However, in Japan, the prevalence of *H. pylori* infection in younger individuals has been declining at an accelerating rate [3] and eradication therapies for *H. pylori* have been approved by the national health insurance in 2013; thus, the incidence of GC has been gradually decreasing [4–6].

Recent advancements in cancer genome sequencing have revealed the comprehensive somatic mutation profiles in GCs. The Cancer Genome Atlas (TCGA) group has reported that GC can be genetically/etiologically classified into four subgroups: genetically stable, chromosomal instability, microsatellite instability, and EBV-associated GCs [7]. Large-scale sequencing studies of GC identified and confirmed frequent gene amplifications in genes encoding receptor tyrosine kinases (e.g., *ERBB2*, *ERBB3*, *EGFR*, *FGFR2*, *MET*, and *VEGFR*) and somatic mutations in *p53*, *ARID1A*, *PIK3CA*, *SMAD4*, *CDH1* (*E-Cadherin*), and *RHOA* genes, with high frequencies in the last two specifically among diffuse-type GC (DGC) [7–12]. Somatic gene fusion of *CLDN18*/*ARHGAP* has also been reported as a frequent event in DGC [7, 13]. These somatic genetics have clarified the molecularly defined subtypes of GC and their driver events, which could be candidate therapeutic targets [14, 15].

Germline variations in the personal human genome are also known to play important roles in carcinogenesis in various organs, including the stomach [16]. Investigation of the cancer incidences among Japanese migrants in Hawaii showed that the rates of GC had decreased in the first- and then second-generation immigrants but were still higher than the rate in the local populations in Hawaii [17], suggesting that individuals of east Asian ethnicity harbor genetic traits predisposing them to GC. Large-scale genome-wide association studies (GWAS) of sporadic GCs have been conducted in east Asian populations and have identified several common germline variations (described later) [18–21]. As to rare variants with large effect sizes, well-known germline variations among hereditary DGC (HDGC) families have been found in *CDH1* worldwide [22] and other variants have also been reported in genes encoding DNA repair machineries (*BRCA1*/*2*, *PALB2*, *RAD51*, *MSH2*, *ATR*, *NBN*, and *RECQL5*) [23, 24]. However, hereditary GCs are clinically difficult to identify in east Asian countries with considerably high incidences of sporadic GCs. Therefore, in these regions, the precise frequency of hereditary GC and the predisposing germline genetics have not been fully elucidated.

Since the early 1960s, statistical epidemiology of cancer patients has revealed various links of lifestyles, occupational environments, and dietary factors to the development of cancers [25, 26]. Investigation of the epidemiological factors that predispose individuals to malignancies have long been warranted in the view of preventive medicine. However, cancers typically arise because of complexed combinations of epidemiological factors, and carcinogenesis occurs over a long duration of time, during which the past epidemiological information of patients may become obscure. Therefore, precise enumeration and evaluation of the epidemiological links between lifestyles and cancer in a scientifically robust manner has been challenging. Genetic analysis so-called “mutational signatures” in the cancer genome has rapidly emerged as a solution to this concern, which would make it feasible to evaluate so-far missing links between epidemiology and carcinogenesis on a genetic basis [27–31].

**Fig. 1:**
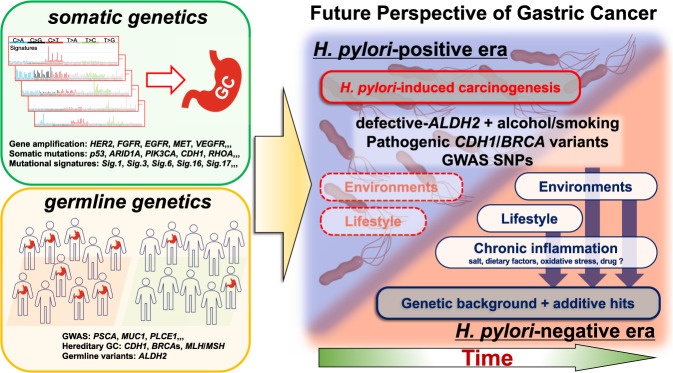
Future perspectives for gastric cancer (GC) in east Asia in the upcoming *H. pylori*-negative era. Schematic summary of this review article. State-of-the-art somatic and germline genetic analyses have clarified the precise molecular pathology of gastric carcinogenesis (left). Based on such knowledge of the genetics of GC in the current era, the future perspectives for new types of GC in the *H. pylori*-negative era are speculated (right). Graphs of the mutational signatures are derived from COSMIC website (Mutational Signatures v2, https://cancer.sanger.ac.uk/cosmic/signatures_v2.tt)

In this review, we first summarize the recent findings regarding the links between lifestyles and somatic/germline genetics in GC patients among east Asian populations, with special focuses on alcohol intake and smoking habits. We also summarize the germline variations that predispose affected individuals to GC, including a recent report of a higher-than-expected frequency of *CDH1* germline variants among Japanese patients with GCs, most of which were clinically considered as sporadic cases [32]. Finally, we discuss the future perspectives of GC and its prevention in the upcoming era of *H. pylori*-negative background (Fig. 1).

## Epidemiology of *H. pylori* and lifestyles in GC

The best-known extrinsic risk factor for GC is *H. pylori* infection worldwide [33, 34]. Although the exact proportion of *H. pylori*-positive GCs including those with past infections is a matter of debate, as is how *H. pylori*-positivity should be evaluated serologically, histologically, and endoscopically [35–39], the incidence of *H. pylori*-positive GC is considered to be extremely high in east Asian countries, such as 96.0% in Korea [35, 36] and 94.6% or higher in Japan [37–39]. A trans-ethnic meta-analysis of 12 studies of 1,228 GCs showed a statistical link between *H. pylori* seropositivity and non-cardia GCs, with an odds ratio (OR) of 3.0 (95% confidence interval [CI], 2.3–3.8), and an even stronger correlation (OR, 5.9; 95% CI, 3.4–10.3) when the duration between blood data for *H. pylori* infection and GC diagnosis was longer than 10 years [40]. Another trans-ethnic meta-analysis of 19 studies with 2,491 GCs also showed an epidemiological link between *H. pylori* seropositivity and GCs of any kinds with an OR of 1.92 (95% CI, 1.32–2.78) [41]. Genetic variants of *H. pylori* strains, specifically those in *CagA* and *VacA* genes, are also known to be linked to differential risks for GC [34, 42, 43]. Dr Hatakeyama’s group reported that east Asian CagA has higher binding affinity (by two orders of magnitude) for the N-SH2 domain of SHP2 than type I western CagA, and such strong binding makes the structure of the N-SH2 more relaxed and more efficiently activates SHP2, leading to the neoplastic transformation of gastric epithelial cells [44]. Given the strong correlation between *H. pylori* and GC, a substantially large portion of GCs has been attributed to *H. pylori* infection and the subsequent pathological inflammation/regeneration of gastric mucosa [33, 34, 42]. However, the prevalence of *H. pylori* infection is predicted to be decreasing in Japan. A meta-analysis showed that the multivariable adjusted prevalence of *H. pylori* infection has been drastically decreasing among younger individuals [3]; the predicted prevalence among populations born in 1920, 1930, 1990, and 2000 was 65.9% (95% CI, 63.9–67.9), 67.4% (95% CI, 66.0–68.7), 15.6% (95% CI, 14.0–17.3), and 6.6% (95% CI, 4.8–8.9), respectively. In addition, the Japanese government broadened the application of *H. pylori* eradication therapy for national health insurance in 2013, and ~1.5 million people in Japan undergo the eradication of *H. pylori* each year [5]. Presumably due to the combinations of such decreasing prevalence of *H. pylori* infection and increase of the eradication therapy as well as the early detection surveillance of GC [45], the incidence of GC in Japan has been gradually decreasing [4–6], and deaths from GC have fallen from 48,427 in 2013 to 45,509 in 2016 [5]. Importantly, this continuously decreasing trend in *H. pylori*-related GC implies the emergence of an era of GCs with *H. pylori*-negative background in the coming decades. Therefore, investigations of the etiology and carcinogenesis of *H. pylori*-negative GC are warranted.

Regarding our daily lifestyles, it has been shown that the intake of salty and smoked foods is related to the development of GC [46]. Like other human malignancies, epidemiological studies have suggested that GC is attributed to alcohol intake and smoking habits [47–49]. A large-scale investigation of 54,682 Japanese population with a 13.4-year follow up revealed that alcohol intake was significantly associated with an increased risk of GC among men, with hazard ratio (HR) as high as 1.85 (95% CI, 1.35–2.53) compared to nondrinkers [47]. This study also showed that every 10-g increase in alcohol intake led a HR of 1.07 (95% CI, 1.04–1.10) for GC in men. Trans-ethnic meta-analyses of the risks of alcohol intake for GC showed that alcohol elevated the risk of GC with an OR of 1.39 (95% CI, 1.20–1.61) (in 19,302 individuals from ten studies) [48] and a risk ratio of 1.17 (95% CI, 1.00–1.34) (in 5,886,792 individuals from 23 studies) [49]. For smoking, multiple meta-analyses confirmed a risk for GC among populations from various ethnic backgrounds [50, 51]. The Stomach cancer Pooling Project, which included 10,290 patients with GC and 26,145 controls, showed that, when compared to never smokers, the OR of current smokers was as high as 1.25 (95% CI, 1.11–1.40), and the OR of individuals with a smoking history longer than 40 years was elevated to 1.33 (95% CI, 1.14–1.54) [50].

## Mutational signatures in the cancer genome

**Fig. 2:**
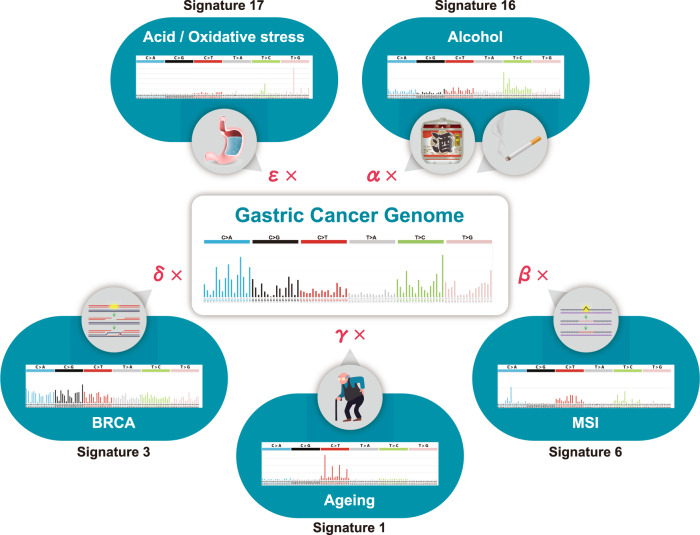
Mutational signatures in the cancer genome. The somatic mutation profile in an individual gastric cancer genome (center) can be mathematically factorized into cumulative combinations of mutational signatures (outer graphs). To date, more than 60 mutational signatures have been proposed by COSMIC, several of which are linked to specific carcinogenic factors (smoking, alcohol use, ultraviolet exposure, ageing, etc.). By calculating the contribution score for each mutational signature (α, β, γ, etc.), the relative contributions of the causative factors to carcinogenesis can precisely be estimated. Graphs of the mutational signatures are derived from COSMIC website (Mutational Signatures v2, https://cancer.sanger.ac.uk/cosmic/signatures_v2.tt)

The cancer genome harbors numerous somatic mutations resulting from DNA damages and erroneous repairs caused by various endogenous and exogenous processes, including mutagenic chemical exposures, physical destruction of DNA, and defective DNA repair pathways, among others. Single-nucleotide substitutions (C>A, C>G, C>T, T>A, T>C, and T>G) can be classified into 96 types according to their neighboring two nucleotides (both 5′ and 3′), and somatic mutations in the cancer genome can be defined as a set of those 96 types of substitutions. Interestingly, analysis of the whole-genome or whole-exome sequences of large cohorts of cancers has revealed that cancer genome mutations can be mathematically factorized into a mixture of patterns based on the combinations of these 96 substitution types (Fig. 2). These patterns of somatic mutations are called mutational signatures (or mutational spectra) [27–31]. A cancer genome is composed of additive (nonnegative) accumulations of the source patterns of the mutational signatures; thus, nonnegative matrix factorization is utilized to compute contribution scores of the mutational signatures in each of the cancer genome [29]. The Catalog of Somatic Mutations in Cancer (COSMIC) group categorized such mutational signatures into 30 types (Mutational Signature v2, March 2015) (https://cancer.sanger.ac.uk/cosmic/signatures_v2.tt). Some of these mutational signatures have been suggested to be epidemiologically linked to specific carcinogenic factors, such as smoking (Sig.4), ultraviolet exposure (Sig.7), alkylating agents (Sig.11), aristolochic acid (Sig.22), aflatoxin (Sig.24), and tobacco chewing habit (Sig.29), while others are etiologically linked to intrinsic factors like ageing (Sig.1), altered activation of AID/APOBEC cytidine deaminases (Sigs.2, 9, and 13), germline/somatic *BRCA* mutations (Sig.3), defective mismatch repair (Sigs.6, 15, 20, and 26), and altered activity of the error-prone polymerase POLE (Sig.10) (Mutational Signature v2, COSMIC). Recently, COSMIC has further extended these signatures into more than 60, including those of possible sequence artefacts (Mutational Signature v3.1, Aug 2020).

By analyzing the proportions of these mutational signatures in the cancer genome, it can be feasible to mathematically enumerate the relative contributions of each factor to carcinogenesis [27–31] (Fig. 2).

## Identification of an east Asian-specific subtype of GC linked by mutational signatures, germline factors, and lifestyles

In a recent report, in which a large cohort of trans-ethnic 531 GCs was classified based on the patterns of the mutational signatures in the cancer genome, a GC subgroup was shown to have a high contribution of Sig.16 [32]. This GC subgroup was characteristic for its Asian ethnicity and inactive *ALDH2* allele (rs671 AA or AG). In a focused investigation of 243-case Japanese GCs, it was revealed that 6.6% (16/243) of cases were classified in a cluster with a high contribution of Sig.16; furthermore, 68.8% (11/16) of these cases were alcohol consumers with inactive *ALDH2* allele (rs671 AA or AG) [32]. In a detailed analysis of the frequencies of the Sig.16 mutations found in each of 243 Japanese GC, patients with both an alcohol use habit and an inactive *ALDH2* allele showed synergistically increased numbers of Sig.16 somatic mutations (11.1-fold) compared to other GC patients. The link between Sig.16 and alcohol use has also been reported for other malignancies [52–54]. Although the molecular mechanism linking alcohol intake and Sig.16 mutations remains to be investigated, various acetaldehyde-derived DNA adducts have been identified [55], which could be a cause of the frequent T>C transitions observed in Sig.16. An intriguing additive effect of smoking on the accumulation of Sig.16 in GCs was also identified, in which the Sig.16 somatic mutations were synergistically increased among overall GC patients with the triple combination of alcohol intake, smoking, and a defective *ALDH2* allele (*p* = 0.0339) [32]. This phenomenon is highly east Asian-specific, based on the specificity of the defective *ALDH2* allele among Asian populations [56]. As discussed above, epidemiology showed that alcohol intake and smoking had significant but only mild associations with GCs [47–51]; however, an integrated investigation of mutational signatures, germline genetics, and lifestyle information showed that these risk factors are apparently more obvious among specific east Asian populations with a defined germline variant. It will also be important to identify any characteristic mutational signatures specifically among non-Asian populations.

## Germline variations that predispose affected individuals to GC

Large-scale GWAS of single-nucleotide polymorphisms (SNPs) have also been conducted to identify susceptibility loci in GC. These studies have been conducted almost exclusively in east Asian populations such as Japanese and Chinese. Comprehensive GWAS by Dr Hirohashi’s and Dr Matsuda’s groups in Japan identified SNPs that were significantly associated with GC. SNPs of *prostate stem cell antigen* (*PSCA*) (8q24.3) and *Mucin1* (*MUC1*) (1q22) were shown to be associated with DGC [18–20] (Table 1). In addition, SNPs on 12q24.11-12 (*cut like homeobox 2* [*CUX2*]), 20q11.21 (a gene cluster of the *defensin beta* family), and 9q34.2 (the *ABO* locus) were also associated with GC [20] (Table 1), and were more strongly associated with DGC than intestinal-type GCs, although it was not statistically significant. The high-risk SNPs of *PSCA* and *MUC1* identified among Japanese populations were also confirmed among Korean populations [18, 19] (Table 1). A GWAS of Chinese GCs showed that multiple variants on 10q23 were significantly correlated with GC, and a notable value was found for rs2274223 in *phospholipase C epsilon 1* (*PLCE1*) [21] (Table 1).

**Table 1:** Representative GC-associated germline variants identified by GWAS

Gene	Chromosome	SNP	Ethnicity	Risk	Association with histology or anatomy	Reference
*PSCA*	8q24.3	rs2976392	Japanese	OR, 1.62 (95% CI, 1.38–1.89) effect allele: A	Stronger association with DGC	# [18]
			Korean	OR, 1.90 (95% CI, 1.56–2.33) effect allele: A	Stronger association with DGC	# [18]
			﻿European descent	OR, 1.88 (95% CI, 1.47–2.43) effect allele: A	Significant in both DGC/IGC	# [61]
		rs2294008	Japanese	OR, 1.58 (95% CI, 1.35–1.85) effect allele: T	Stronger association with DGC	# [18]
			Korean	OR, 1.91 (95% CI, 1.57–2.33) effect allele: T	Stronger association with DGC	# [18]
			Chinese	OR, 1.20 (95% CI, 1.15–1.28) effect allele: T	Analysis of non-cardia GC	# [58]
			﻿European descent	OR, 1.88 (95% CI, 2.42–7.70) effect allele: T	Significant in both DGC/IGC	# [61]
			Caucasian	OR, 1.42 (95% CI, 1.23–1.66) effect allele: T	Particularly in non-cardia GC	# [62]
			﻿Latin American	OR, 2.34 (95% CI, 1.36–4.01) effect allele: T	Significant in both cardia/non-cardia GC significant in both DGC/IGC	# [63]
*MUC1*	1q22	rs2070803	Japanese/Korean	OR, 1.71 (95% CI, 1.48–1.98) effect allele: G	Stronger association with DGC	# [19]
		rs4072037	Japanese/Korean	OR, 1.66 (95% CI, 1.44–1.93) effect allele: A	Stronger association with DGC	# [19]
			Chinese	OR, 0.75 (95% CI, 0.67–0.84) effect allele: G	Significant in both cardia/non-cardia GC	# [21]
			Chinese	OR, 0.75 (95% CI, 0.69–0.79) effect allele: G	Analysis of non-cardia GC	# [58]
			Chinese	OR, 1.33 (95% CI, 1.22–1.45) effect allele: A	–	# [59]
			Korean	OR, 0.82 (95% CI, 0.72–0.94) effect allele: G	Not different between DGC/IGC	# [65]
			East Asian/Chinese/Korean	OR, 0.76 (95% CI, 0.69–0.84) effect allele: G	Significant in both cardia/non-cardia GC	# [60]
			﻿European descent	OR, 0.64 (95% CI, 0.49–0.81) effect allele: G	Significant in both DGC/IGC	# [61]
*CUX2*	12q24.11-12	rs6490061	Japanese	OR 0.905 (*P* = 3.20 × 10^−8^) effect allele: T	Not statistically different between DGC/IGC	# [20]
*DEFB cluster*	20q11.21	rs2376549	Japanese	OR 1.109 (*P* = 8.11 × 10^−10^) effect allele: C	Not statistically different between DGC/IGC	# [20]
*ABO locus*	9q34.2	rs7849280	Japanese	OR 1.148 (*P* = 2.64 × 10^−13^) effect allele: G	Not statistically different between DGC/IGC	# [20]
*PLCE1*	10q23	rs2274223	Chinese	OR 1.31 (*P* = 8.40 × 10^−9^) effect allele: G	Significantly associated with cardia GC	# [21]
			Korean	OR, 0.96 (95% CI, 0.87–1.06) effect allele: G		# [65]
			Japanese	Not significant		# [20]
			Caucasian	Not significant		# [64]
			﻿European descent	Not significant		# [61]

The molecular biological functions of these SNPs have been investigated, and the cancer-related functional disturbances they presumably cause have been described. For instance, *PSCA* is confirmed to be expressed in gastric epithelial cells, and substitution of a C allele with the high-risk T allele at rs2294008 in the 1st exon reduced its transcriptional activity [18]. It is also noteworthy that the alleles of this SNP of *PSCA* have opposing effects in GC and duodenal ulcers [57]. The T allele, which results in a longer membranous PSCA, has growth-promoting effects in inflamed gastric epithelia, making the T allele a high-risk factor for GC. In contrast, the C allele, which results in a shorter cytosolic PSCA, may enhance the immune reaction and might accelerate pathological inflammation in the duodenum as well as induce antitumor immunity during gastric carcinogenesis [57]. It has also been reported that the rs6490061 SNP in the *CUX2* significantly reduced its mRNA expression in response to *H. pylori* infection [20]. Additional cell biological experiments investigating the effects of the identified SNPs through gene editing using CRISPR or other techniques would help reveal precise molecular mechanisms underlying the significant predispositions to GCs in affected individuals, which may reveal novel modality of preventive strategies against GCs.

These polymorphisms, which were initially identified in Japanese and/or Chinese populations, have been confirmed in other studies of not only other east Asians [58–60] but also western populations, including Caucasians [61–63] (Table 1). However, at the same time, discrepancies have also been reported in the significance of these SNPs among populations of different ethnic backgrounds [64]. For instance, the significant association of the *PLCE1* polymorphism with GC identified among Chinese populations has not been confirmed among Caucasian populations [64], and in fact, an inverted trend of correlation was observed in a Korean population, although not statistically significant [65] (Table 1). The abovementioned GWAS from Japan did not find the *PLCE1* SNP to be significant [18–20]. PLCE1 is a phospholipase enzyme that connects signals from small GTPases to various pathways, including MAP kinase cascades [66–68]; thus, it may regulate cancer-related processes, such as cell growth and differentiation. This *PLCE1* SNP (rs2274223) was initially identified as a common risk allele for both esophageal and GCs [21], and a meta-analysis of Chinese populations reported that it is associated with cardia GC [69]. The prevalence of cardia and non-cardia GCs is known to differ substantially between ethnicities, and in Japan, non-cardia GC has been characteristically prevalent [70, 71]; thus, the inconsistency in the significance of the *PLCE1* SNP between Chinese and Japanese GCs might reflect the difference in the preferred location of GC as well as its background environmental factors.

From the viewpoint of preventive medicine, with the relatively low ORs and small effect sizes of the common germline variants in GCs identified thus far, genotyping of these SNPs alone is not sufficient to stratify individuals for the prediction of gastric carcinogenesis. Thus, a high-risk approach using a small number of common variants may be insufficient to effectively prevent GC.

## Unexpectedly high incidence of germline variants in *CDH1* among east Asians with GC

**Fig. 3:**
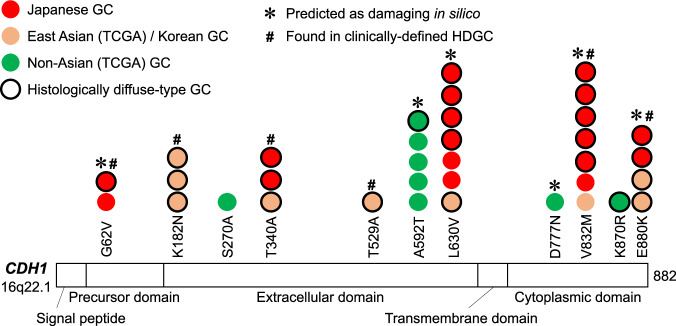
Germline variants in CDH1 gene identified in GC patients. A histogram of non-silent germline variants of *CDH1* gene identified in a recent trans-ethnic study [32]. Colors of the circles represent ethnicities of the patients as indicated. Circles with black rims represent cases of DGC. * and # represent *CDH1* variants that were predicted as damaging in silico and found in clinical HDGC families, respectively

In addition to the germline genetics described above, HDGC is a well-known GC-predisposing syndrome that is attributed to germline variants of causative genes such as *CDH1* [22]. CDH1 is a member of cadherin superfamily consisting of a precursor domain, five extracellular cadherin domains, a transmembrane domain, and a cytoplasmic domain (Fig. 3) and plays an important role in the epithelial cell-to-cell adhesion [72]. Its loss-of-function is known to contribute to the disseminative and invasive phenotypes of cancer cells by affecting various molecular pathways [72]. Germline variants in other genes have also been found in DNA repair machinery (*BRCA1*/*2*, *PALB2*, *RAD51*, *MSH2*, *ATR*, *NBN*, and *RECQL5*) [23, 24], as described later. The *CDH1* germline variants among HDGC was first discovered in Maori kindred in New Zealand in 1998 [73]. Thus far, at least 155 germline *CDH1* variants have been identified worldwide [74–76], and recently, the International Gastric Cancer Linkage Consortium (IGCLC) updated the diagnostic criteria and clinical practice guidelines for HDGC (Aug 2020) (https://hereditarydiffusegastriccancer.org/) [22].

However, most of the *CDH1* variations were reported among non-Asian populations. As high as 40% of detection rates of germline *CDH1* variants have been reported among non-Asian HDGC families [77–80], although one report of European familial GC identified no germline variants in *CDH1* [81]. In contrast, the results of several representative studies of germline *CDH1* variants in cases of possible HDGC or familial GC in Japan [82–86] suggested that the detection rates of *CDH1* variants among east Asian populations are relatively rarer (Table 2). Thus, determining the frequency of germline *CDH1* variants in familial GCs among east Asians has remained elusive, due in part to the paucity of comprehensive studies with large cohorts of HDGC families as well as the difficulty in identifying genuine cases of HDGC among east Asians because of the high incidence of sporadic GC.

**Table 2:** Representative studies of germline *CDH1* variants in familial GCs among trans-ethnic populations

Ethnicity/country	Frequency of germline CDH1 variants		Criteria of familial GC	Mutaiton		Reference
Northern European desscents, and Spanish, Haida, French-Canadian, and Italian descents, including unknown origin	31.0%	13/42 families	Two or more DGC in first-degree relatives, with at least one diagnosed before 50 Two or more GC, with at least one DGC diagnosed before 50 Three or more DGC in first-degree relatives Three or more GC with at least one DGC Individuals diagnosed with DGC at younger than 45 Individuals diagnosed with both DGC and LBC One family member diagnosed DGC and another with LBC One family member diagnosed DGC and another with colon cancer	382delC	Frameshift	[77]
				G892A	A298T	
				1064insT	Frameshift	
				1134 del8, ins5	Deletion	
				1212delC	N405I*fs*	
				T1226C	W409R	
				1476delAG	Frameshift	
				1779insC	Frameshift	
				2061delTG	Frameshift	
				G2195A	R732A	
				2310delC	Frameshift	
				IVS5(+1) G>A	Splice site	
				IVS11(+5) G>A	Splice site	
English, Irish, Spanish, Columbian, Filipino, Swedish/Norwagian, including unknown origin	39.5%	15/38 families	at least 2 GCs with 1 DGC younger than 50 either 1 DGC younger than 35 or multiple DGCs older than 50	283C>T	Q95X	[78]
				715G>A	G239R	
				1137G>A splicing	Splicing site	
				1397-1398delTC	Frameshift	
				1682-1683insA	Frameshift	
				1901C>T	A634V	
				1913G>A	W638X	
				2064-2065delTG	Frameshift	
				2164+5G>A splicing	Splicing site	
				2195G>A	R732Q	
				2245C>T	R749W	
				2343A>T	E781D	
				2398delC	Frameshift	
Caucasian, Hispanic, Maori, Chinese (one family)	29.2%	7/24 families	IGCLC criteria	49-2A>C	Splice site	[79]
				353c>G	T118R	
				715G>A	G239R	
				1107delC	Frameshift	
				1137G>A	Splice site	
				1391_1392delTC	Frameshift	
				1901C>T	A634V	
				2095C>T	Q699X	
				2440-6C>G	Splice site	
Netherlands, Portugal, Germany, Italy, Poland	0.0%	0/53 families	Diagnosed below 35 families with two GCs at or below 60 Families with three GCs at or below 70			[81]
Japanese	0.0%	0/14 families	At least three relatives had GCs with at least one first-degree relative of the other two At least two successive generations had GCs			[84]
Japanese	2.6%	2/78 families	Three and more GCs in a family At least two successive generations had GCs GCs diagnosed younger than 50 in one of the relatives	1243A>C	I415L	[85]
Japanese	11.8%	2/17 individuals	At least one sibling diagnosed with GC	IVS+6T>C	Splice site	[86]
				2494G>A	V832M	
Japanese	15.4%	2/13 families	IGCLC criteria	1212delC	N405I*fs*X12	[82]
				164-?_387+?del	V55G*fs*X38	

Representative studies, which investigated more than 10 kindreds of HDGC or familial GCs, are listed

The disease penetrance of the germline *CDH1* variants had been estimated to be substantially high among non-Asian populations, and one study concluded that the cumulative risk of GC by 80 years of ages was 67% (95% CI, 39–99) and 83% (95% CI, 12–84) in men and women, respectively [87], and another estimated these risks as 40% (95% CI, 12%–91%) and 63% (95% CI, 19%–99%) by the age of 75 years for men and women, respectively [78]. However, the disease penetrance among east Asian populations has not been established to date.

To evaluate the actual germline contributions to unselected GCs in east Asian populations, the germline genetics of the abovementioned 243-case Japanese GC cohort were investigated [32]. Analysis of 624 cancer-related genes revealed that the *CDH1* gene had the highest density of germline rare variants (ratio of variant frequency to the length of the gene) [32]. In total, 18 out of the 243 Japanese GCs (7.4%) harbored germline variations in *CDH1*. All the non-silent germline variations identified in the study are listed in Table 3 and shown in Fig. 3. Most of the Japanese GCs with *CDH1* variations (77.8%, 14/18 cases) were diagnosed as DGC (Table 3) [32], which is consistent with the molecular dysfunction of CDH1 specifically in DGC [88] and strongly suggests pathogenic roles for these variants in the gastric carcinogenesis. For DGC, the frequency of *CDH1* variants in this cohort (13.3%, 14/105 cases) was 4.1-fold higher than that in non-Asian GCs of TCGA (3.2%, 2/62 cases) [32]. Distributions of the variants in *CDH1* gene among Japanese GCs showed that they were enriched at five positions that were shared with Korean populations with GC [89] but were clearly different from those of non-Asians (Table 3) (Fig. 3). Four of the five have previously been reported in early onset GCs in Korea [89] and families with GCs in Japan and Brazil, including p.G62V in a family of Japanese ethnicity [83, 86, 90]. Two of them, T340A and V832M, were suggested to be pathogenic based on in vitro experiments [91, 92]. Molecular links between the *CDH1* variants and gastric carcinogenesis specifically in relation to the differences in ethnic backgrounds should be investigated further in the future.

**Table 3:** Germline *CDH1* variants identified in the 243-case Japanse and trans-ethnic GCs in a recent report

*CDH1* variants	Exon	Domain	HDGC^a^		ClinVar	Polyphen2 (HumDiv)	Japanese 243 GCs^b^			Korean population^c^		TCGA non-Asian 212 GCs	Japanese 105 DGCs	TCGA non-Asian 62 DGCs	DGC: Japanese vs. non-Asian	Japanese ToMMo 1070 individuals	Japanese DGC vs. ToMMo	1000 Genomes EAS 504 individuals	1000 Genomes EUR 503 individuals	1000 Genome EAS vs. EUR
							Age	Lauren’s classification	Family history of cancers	Age	Lauren’s classification									
**p.G62V**	**Exon 3**	Precursor region	**◯**	Ref. # [83]	Uncertain significance	Probably damaging	**80s**	IGC	**Yes**				**1**			**3**				
							**60s**	**DGC**	**No**											
**p.K182N**	**Exon 5**	Extracellular domain	**◯**	Ref. # [89]	Conflicting interpretations of pathogenicity	benign				**30s**	**DGC**					**1**		**1**		
										**40s**	**DGC**									
										**50s**	**DGC**									
**p.S270A**	**Exon 6**	Extracellular domain			Conflicting interpretations of pathogenicity	benign						1								
**p.T340A**	**Exon 8**	Extracellular domain	**◯**	Ref. # [89, 90, 92]	Benign	benign	**80s**	**DGC**	**Yes**				**2**			**6**		**1**		
							**60s**	**DGC**	**No**											
										**30s**	**DGC**									
**p.T529A**	**Exon 11**	Extracellular domain	**◯**	Ref. # [89]	Conflicting interpretations of pathogenicity	benign				**30s**	**DGC**									
**p.A592T**	**Exon 12**	Extracellular domain			Benign	Probably damaging						5		1					2	
**p.L630V**	**Exon 12**	Extracellular domain			Benign	Probably damaging	**70s**	**DGC**	**Yes**				**4**			**9**		**6**		
							**70s**	**DGC**	**No**											
							**80s**	IGC	**Yes**											
							**60s**	**DGC**	**No**											
							**80s**	**DGC**	**Yes**											
							**60s**	IGC	**Yes #**											
										**60s**	**DGC**									
**p.D777N**	**Exon 15**	Cytoplasmic domain			Conflicting interpretations of pathogenicity	Probably damaging						1								
**p.V832M**	**Exon 16**	Cytoplasmic domain	**◯**	Ref. # [86, 92]	Benign	Probably damaging	**90s**	**DGC**	**No**				**6**			**10**		**2**		
							**50s**	**DGC**	**Yes**											
							**50s**	**DGC**	**Yes**											
							**60s**	IGC	**No**											
							**60s**	**DGC**	**No**											
							**50s**	**DGC**	**Yes #**											
							**60s**	**DGC**	**Yes**											
										**70s**	IGC									
**p.K870R**	**Exon 16**	Cytoplasmic domain			Not found	benign						1		1						
**p.E880K**	**Exon 16**	Cytoplasmic domain	**◯**	Ref. # [89]	Conflicting interpretations of pathogenicity	Probably damaging	**30s**	**DGC**	**Yes**				**1**			**7**		**2**		
										**30s**	**DGC**									
										**30s**	**DGC**									
							**18 (7.41%)**					8 (3.77%)	**14 (13.33%)**	2 (3.23%)	**4.13-fold**	**36 (3.36%)**	**3.96-fold**	**12 (2.38%)**	2 (0.40%)	**5.99-fold**

Germline CDH1 variants discovered in a recent report [Ref. #^32^] are listed. Mixed type GCs in the Lauren classification were categorized as DGC

*DGC* diffuse-type GC, *IGC* intestinal-type GC, *EAS* east Asian, *EUR* Europian populations

Lauren’s classification (if DGC), family history (if Yes), and GC case numbers among east Asians are highlighted as bold.

^a^Reported in GC cases that met clinical criteria of HDGC such as strong femilial aggregation and/or extremely early onsets [Ref. #^83^, ^86^, ^89^, ^90^, ^92^]

^b^Data from a recent study of large-scale trans-ethnic GCs [Ref. #^32^]

^c^Combined data from TCGA (Korean) and a large-scale Korean study of early onset GCs [Ref. #^89^]

It should be noted that the combined frequency of *CDH1* rare variants among Japanese DGCs is 3.96-fold higher than that of general Japanese population (3.4%, 36/1,070). Furthermore, in the first place, it was firstly documented that as high as 3.4% of the general Japanese population harbors pathogenic germline *CDH1* variants [32], which has significant impacts in clinical fields, as discussed below. However, this observation needs to be confirmed by an independent analysis with a larger cohort. As to V832M, a negative study was recently reported in a Korean population [93].

These germline *CDH1* variants had not been identified as pathogenic in any previous GWAS or other large-scale genetic studies [18–21, 89]. One possible reason for this is the low minor allele frequency of the variants. The frequency of even the most common germline *CDH1* variant found in the ToMMo database, V832M, is only 0.93%; thus, the *CDH1* rare variants had probably been omitted from previous GWAS due to their insufficient statistical power. Recent statistical methods to analyze rare variants such as SKAT (SNP-set (Sequence) Kernel Association Test) where sets of rare/common variants, for instance in a gene or a region, can be evaluated integratively [94, 95] might make it feasible to discover significant rare variants among GC including those of *CDH1*. Another reason could be related to the ways of curation in the discovery of pathogenic rare germline variants; germline *CDH1* variants were not intensively focused unless they were clearly annotated by the ClinVar database [96] or if they existed at measurable frequencies in general populations. None of the *CDH1* variants in Table 3 are currently annotated as “pathogenic” in the ClinVar database.

Four out of the five condensed germline variants of *CDH1* specifically among Japanese GCs were also shared in Korean populations with GC [32, 89] (Table 3), indicating that these germline variants of *CDH1* are specifically and widely distributed among east Asian populations. Thus, it is hypothesized that common ancestral events in multiple loci of the *CDH1* gene can explain the increased incidence of GC in east Asia; however, their evolutional significance, including any possible benefit, is still unclear. Interestingly, one of the oldest modern humans, a 45,000-year-old male individual found in Ust’-Ishim in Siberia [97], harbored a heterozygous V832M (rs35572355 G>A) germline variant in *CDH1* (https://bioinf.eva.mpg.de/jbrowse). The Ust’-Ishim genome shared more alleles with modern east Asian populations among non-African populations [97]; thus, germline *CDH1* variants can be assumed to be rooted at least in that era.

## Germline variants of *BRCA* family genes and GC

Germline variations in the genes encoding double-strand break repair machinery, such as *BRCA1*/*2*, *PALB2*, and *RAD51*, have been shown to be causative factors for familial GCs [23]. Variations in other DNA repair genes, such as *ATR*, *NBN*, and *RECQL5*, and the mismatch repair gene *MSH2* have also been reported in cases of HDGC without *CDH1* variations [24]. It is generally difficult to extract pathogenic rare variants from many other nonpathogenic backgrounds because a statistical approach is not usually effective. In addition, unlike breast and gynecological cancers, there have been few reports of systematic germline surveys in large-scale GC cohorts. In the study of a Japanese GC cohort mentioned above, 9.1% of the Japanese GC patients (22/243 cases) had probably pathogenic germline variants with functional defects in BRCA pathways, i.e., cases with the BRCA-related somatic mutational signature (Sig.3) [32]. This frequency is comparable to that among non-Asian GCs in the TCGA data (9.0%, 19/212 cases) [32]. These frequencies differ slightly from those reported in other large-scale genetic studies. For instance, the prevalence of pathogenic variants in *BRCA* pathway and *TP53* genes were reported to be 5.7% in Japanese patients with breast cancer [98], and pathogenic *BRCA1*/*2* variants were detected in 12.1% and 12.7% of western European and Asian patients with breast and ovarian cancers, respectively [99]; however, GCs were not investigated in these studies and different criteria were utilized for mutation detection. It has been proposed that malignancies with the BRCA-related mutational signature are good candidates for PARP inhibitors in combination with platinum-induced DNA damage [100], which might be clinically applicable for GC patients.

## Clinical intervention for hereditary GC

It is of worth noting that, in the abovementioned study [32], the germline *CDH1* variants found in east Asian populations exhibited mild disease penetrance in affected individuals. Out of the 18 Japanese GC individuals with germline *CDH1* variants, 11 had family histories of cancers (Table 3), and one had a lobular breast carcinoma, consistent with a germline variant of *CDH1* [22]. However, only one individual fulfilled the IGCLC criteria for HDGC, as a DGC was diagnosed in the 30s of age [32]. The seven other individuals did not have any family history of malignancy. Thus, Japanese individuals even with possibly pathogenic *CDH1* variants do not always develop GC. The reason why the disease penetrance of the *CDH1* variants among Japanese is lower is not scientifically obvious to date. A possible reason for this might be because gastric carcinogenesis requires additional etiological hits that may be missing in the current lifestyles of east Asians. A recent case report from Japan investigated familial GC in two siblings; one sibling was infected with *H. pylori* and had an advanced GC, while the other was free from *H. pylori* and had an early-stage GC [101]. Although additional larger studies are necessary to draw a conclusion, this suggests that *H. pylori* infection plays a significantly additive role in gastric carcinogenesis, even in individuals with pathogenic germline *CDH1* variants. It is also speculated that the disease penetrance of HDGC in east Asian populations is going to be modified due to yet undefined and ever-changing lifestyle factors, even without *H. pylori* infection in the upcoming *H. pylori*-negative era.

The updated ICGLC guidelines (Aug 2020) for HDGC has recommended prophylactic gastrectomy for individuals with pathogenic *CDH1* variants [22]. However, as discussed above, the disease penetrance and behaviors of *CDH1* variants are substantially different between Asian and non-Asian ethnic backgrounds. Therefore, more careful consideration of prophylactic surgery is recommended for individuals of east Asian ethnicity who are just uncertainly suspected as familial GC than for Caucasians [22, 102–104]. A more specific and practical definition of HDGC among east Asian populations, with evaluations of the pathogenicity and influence of lifestyle/environmental factors on the penetrance of *CDH1* variants, including their future trends, is needed.

As has also been discussed by the IGCLC group [22], routine endoscopic surveillance of the stomach is another possible method to prevent the development of advanced GC in the affected individuals. In fact, histologically detectable cancer foci have been discovered by endoscopic multiple sampling in a large portion of individuals with germline *CDH1* variants [105–107], and one study succeeded in identifying HDGC based on endoscopic observations [108]. Advancements in technologies for high-definition endoscopy, including fluorescent techniques, Raman spectrometry, and artificial intelligence [109–113], will hopefully provide increasing evidence that periodic endoscopic surveillance of pathogenic *CDH1* variant carriers is an adequate preventive option for those at risk of GC. Recent advancements in liquid biopsy such as detecting cell-free DNA and circulating tumor cells would also help identify and monitor early-stage GC [114–116]. Thus, prophylactic surgeries would be considered only when genuine high-risk individuals could be identified based on the findings of future research on HDGC.

## Future perspectives

In accordance with the changes in our daily lifestyles and environmental factors in the coming decades, the incidence and epidemiology of GCs in east Asia will also be gradually changing. The recent decrease in *H. pylori* infection among populations such as in Japan [3–6] will greatly impact the epidemiology of GC. It is scientifically hard to speculate the precise molecular pathology of future GCs. As a hypothesis, we postulate that GCs in the *H. pylori*-negative era will be found only among individuals with specific genetic backgrounds combined with risky lifestyles. Individuals with pathogenic germline variants may have risks of developing GC, even without *H. pylori* infection, possibly due to causative lifestyle and dietary factors that induce chronic gastritis, such as intake of salty and smoky foods, smoking, oxidative stresses, and chemical agents that modulate host immunity, as well as other yet unidentified factors [117–120]. Currently, with the high prevalence of *H. pylori*-induced GC, the etiological effects of other lifestyle/dietary factors have been masked in epidemiological studies. However, in the upcoming *H. pylori*-negative era, previously undefined lifestyle and environmental factors related to GC, combined with germline backgrounds, might be manifested more clearly (Fig. 1).

Based on our current knowledge of the genetics of the GC development, the relative frequency of GC with high Sig.16 contributions among individuals with alcohol use/smoking habits with an inactive *ALDH2* allele will likely increase among east Asian populations. The risk of GC among such populations is substantially lower compared to the risk of esophageal cancers, in which smoking and alcohol use combined with *ALDH2*/*ADH1B* risk alleles has an OR of 189.26 (95% CI, 95.1–376.6) [121]. However, when considering a high-risk approach for the prevention of future GC, it is epidemiologically important to reduce the risky daily habits of alcohol use and smoking, especially for east Asian individuals with germline variants of *ALDH2*.

It should be underscored that a higher-than-expected frequency (3.4%) of germline *CDH1* variations among the general Japanese population was recently documented; moreover, enrichment of these variants among Japanese patients with DGC was essentially higher (13.3%) [32]. Thus far, the relative contribution of the germline *CDH1* variants to the overall prevalence of GC in east Asians had been considered to be lower than that in other ethnic groups. However, the frequencies of such GCs among east Asians with genetic background will probably rise in the coming era of *H*. *pylori*-negative GC in combinations with changes in yet undefined lifestyles that would function as additive hits in gastric carcinogenesis. Thus, in the next *H. pylori*-negative era, it can be predicted that genetic predispositions of GC would be revealed more clearly, with the aid of the advancements in statistical methods. In clinical fields, it is necessary to consider the possibility of germline variants in patients with GC in daily practice, even when they are clinically considered sporadic cases. Establishment of practical endoscopic and liquid biopsy strategies to discover early-stage HDGCs are also needed.

The definitive clinical features of *H. pylori*-negative GCs among east Asian populations have not been established due to the difficulties in identifying truly *H. pylori*-negative cases in east Asian countries. Previous studies have suggested some characteristics of *H. pylori*-negative GCs in east Asia, including early diagnosis (often under 60 years of age); more frequently located in the cardia (although controversial); more advanced TNM stage and poorer prognosis; and a higher proportion of DGC and signet-ring cell carcinoma, although these were not always statistically significant [35, 36, 39]. As to TCGA classification of GCs [7], MSI and EBV GCs might arise by etiologies independent of *H. pylori*, although it is worth noting that EBV is known to activate *H. pylori CagA* via inhibition of host SHP1 [122]. Thus, these GCs may be more prevalent among *H. pylori*-negative GCs; however, they will benefit from immune checkpoint inhibitors [123], due to the higher neo-antigen or viral antigen burdens.

In this decade, it is necessary for researchers to extensively characterize *H. pylori*-negative GCs in preparation for its global impact in the upcoming era. Through precise investigations of the somatic and germline genetics of GCs, along with stratifications according to patients’ lifestyles, as shown in this review, it should be feasible to clarify robust, personalized molecular mechanisms of both the current and novel types of GC in the upcoming *H. pylori*-negative era.
